# A Review of Trend of Nursing Theories related Caregivers in Korea

**DOI:** 10.2174/1874434601812010026

**Published:** 2018-02-08

**Authors:** Sung Hae Kim, Yoona Choi, Ji-Hye Lee, Da-El Jang, Sanghee Kim

**Affiliations:** 1 College of Nursing, Graduate School, Yonsei University, Seoul, Korea; 2 College of Nursing, Graduate School, Yonsei University, Samsung Medical Center, Seoul, Korea; 3 College of Nursing, Graduate School, Yonsei University, Severance hospital, Seoul, Korea; 4 College of Nursing, Mo-Im Kim Nursing Research Institute, Yonsei University, Seoul, Korea

**Keywords:** Caregivers, Informal caregivers, Nursing theory, Quality of life, Long-term care, Patient care

## Abstract

**Background:**

The prevalence of chronic diseases has been rapidly increased due to population aging. As the duration of care needs increase, the caregivers’ socioeconomic burdens have also increased.

**Objective:**

This review examines the attributes of caregiving experience and quality of life of caregivers in Korea with a focus on the application of nursing theory.

**Method:**

We reviewed studies on caregivers’ caring for adult patients published till 2016 in 4 bio-medical research portal websites or data bases. A total of 1,939 studies were identified through the keyword search. One hundred forty five studies were selected by a process; of which, 17 studies were theory-applied. Selected studies were analyzed in accordance with the structured analysis format.

**Results:**

Quantitative studies accounted for 76.6%, while 22.1% were qualitative studies and 1.3% were triangulation studies. Caregiver-related studies increased after 2000. Most frequently, the caregivers were spouses (28.4%), and most frequently, care was provided to a recipient affected by stroke (22.5%). The 17 theory-based studies described 20 theories (70% psychology theories, 30% nursing theories). The most frequent nursing theory was the theory of stress, appraisal and coping.

**Conclusion:**

This study sought to better understand caregiving through the analysis of Korean studies on the caregiving experience and caregivers’ QOL and this finding helped presenting empirical data for nursing by identifying the nursing theories applied to the caregiving experience and caregivers’ QOL. The results suggest that the need for further expansion of nursing theories and their greater utilization in the studies of caregiving.

## 1 INTRODUCTION

With the rapid development of medical technology and extension of life expectancy, the number of individuals living to an older age has been increasing globally. According to the Organization for Economic Cooperation and Development [1], the proportion of people aged 65 years or over increased from below 9% in 1960 to 15% in 2010, and this is forecasted to reach 27% by 2050. The transition to an “aging” society has been a direct cause of an increase in the prevalence of chronic diseases, such as cancer, diabetes, neurodegenerative disease, cerebrovascular disease and cardiovascular disease. The diverse complications of prolonged chronic diseases limit the independent daily living of disease sufferers. As the duration of care and care requirements increase, the caregivers’ socioeconomic burdens have also increased. Caregivers mean that individuals providing care to a patient without compensation, such as spouses, family members, friends, or neighbors. Caregivers care for the patient’s health and daily living, providing general physical, mental and emotional support and also, financial assistance [2]. While caregivers were mostly spouses in the past, increasingly, young adults especially female family member have been assuming the role of caregivers for aging parents suffering from chronic disease [3, 4].

Providing care without any compensation requires physical work and dedication and frequently involves pain and loss. Although caregivers endure depression and physical fatigue, they experience satisfaction, becoming accustomed to repetitive caring behaviors, and increased interest in their health management role [5, 6]. For patients in need of diverse physical, mental, and emotional support, above all, caregivers provide a crucial social support that can have a significant effect on the disease process; the importance of caregivers has been emphasized because of the positive effects of caring for patient [7]. However, as a patient’s physical dysfunction becomes more developed, care requirements and dependency increase, leading to a heavier caregiver burden. Under a poor social support system, caregivers more acutely feel the increase in caregiving burden and experience negative emotions [8, 9]. In addition, if the patient was their spouse or of older age than caregivers, stress, depression and burden are more likely to occur [8, 10]. Disease is not solely a patient’s personal problem, rather, it is a complex issue, bringing extreme health problems and continuous burden of medical expenditures on caregivers, which frequently result in conflictual family roles, changes in family function, dysfunction at work and social alienation. In Korea since 2008, the government has provided long-term care insurance and medical benefits and partial caregiving support; however, the number of beneficiaries has been limited by regulation. In Korea, many patients with chronic disease are being taken care of at home, instead of being hospitalized in medical institutions. There are strong social and moral obligations to care for parents because of the family-centered social structure and the backdrop of traditional Confucianism. It is one aspect of the Asian family-centered culture, reflecting morally desirable values.

Caregiving experiences are shaped by the dynamic pattern of interactions with the patients; it cannot be explained by a single attribute. The caregiving experience varies greatly depending on the severity of the disease, the relationship with the patients and the social/economic/cultural environments. In Korea, caring for a sick family member is deemed natural. In the family-centric culture, Korean people are often forced to take a caregivers’ role regardless of their intention. Korean caregivers tended to think of themselves first as a family member (*e.g.*, spouse of husband/wife, the child of their parents), rather than as an independent individual. Also they view their adaptation to disease process as a natural process, rather than as a challenging process [11]. Therefore, there is a need for a theoretical approach to explain the caregivers’ caring phenomenon that derives from the social and emotional cultural contexts of “family caring.” The caring phenomenon cannot be explained by grand theory, which is highly abstract, but may possibly be explained by middle-range nursing theory, which has more concrete concepts, or by applying theories from other fields of study. Even so, there has been no integrative review of nursing theory that explained the Korean caregivers’ experience and life; none of the diverse theories developed in Western countries have been applied to or explained Korea’s unique caring culture. In recent years, the emergence of evidence-based nursing practice has led to a significant increase in the number of quantitative studies, including interventional studies; however, there have been few studies on nursing theory.

This study investigated caring phenomenon through an integrative review of nursing theories described in Korean studies of caregiving experience and quality of life (QOL) of caregivers. The goal of the study was to find a strategy for the development of nursing interventions, based on nursing theories customized for Korea, to improve caregivers’ QOL. We also aimed to identify the grounds for the selection of nursing theories appropriate to the Korean culture of caring. Furthermore, it will also expand knowledge regarding nursing knowledge.

## 2 MATERIAL AND METHODS

### 2.1 Design

This study was a literature review of the application and interpretation of nursing theory in Korean studies on caregiving experiences and caregivers’ QOL, and of research patterns and trends in caregiver-related theories.

### 2.2 Search Strategy and Eligibility Criteria

This study was performed in accordance with the Cochrane Systematic Review Handbook Guidelines [12]. The literature search was conducted using Internet databases, from March 17 to 23, 2016. The databases searched included Research Information Sharing Service (RISS), National Discovery for Science Leaders (NDSL), Korean Studies Information Service System (KISS) and DBpia. The search keywords, compiled using a combination of Korean and English, consisted of the research subject (caregiver, patient family, patient caregiver, patient spouse, family caregiver) and outcome variables (caregiving experience, caregiving appraisal, QOL, nursing theory). Caregiving experience- and QOL-related studies were all included among caregivers and patient family. Studies published in Korean up to 2016 were targeted.

Inclusion criteria of an integrative review were: (a) studies on caregivers of adult patients (19 years or older); (b) studies examining the caregiving experience or caregivers’ QOL; (c) published scientific articles or dissertations; (d) published in either Korean or English.

### 2.3 Selection of Studies

**Fig. (1):**
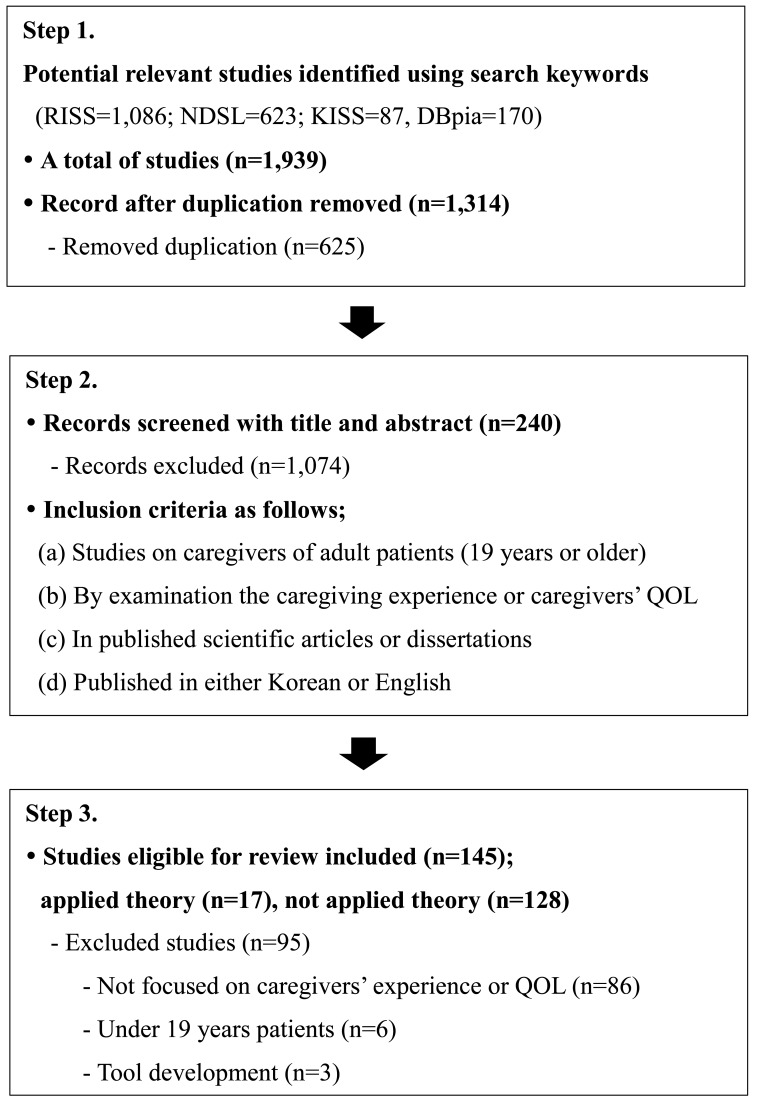
Flow chart for studies selection.

The studies identified through the keyword search were summarized using EndNote. A total of 1,939 studies were identified through the keyword search. After excluding duplication studies, 1,314 studies were left. As a results of the initial screening, 240 studies were selected for full-text assessment by eligibility. The full texts were analyzed, and a total 145 studies were selected for literature review, including 128 studies about caregiving experience and caregivers’ QOL, and 17 studies were based on the theory of nursing or other disciplines (Fig. **1**).

Data extraction was independently carried out by four researchers. To ensure accuracy and consistency in the data collection, the search strategy was agreed upon at two research group meetings. The review and selection of studies were performed by two researchers. Disagreements were arbitrated by another member of the research team.

### 2.4 Data Analysis

Based on the framework for analysis [13], we performed the analysis using a coding sheet and the following keywords: author, publication year, journal, research design, subject disease and nursing theory. For research design, the studies were categorized as qualitative or quantitative (specifically, as experimental or survey studies). In those studies which were not included, the theory of nursing or other disciplines, patients–caregivers relationship, the type of patient disease, and application of a conceptual framework were additionally examined.

## 3 RESULTS

### 3.1 Research Characteristics

Among 145 studies selected for review, patient’s disease type and the nature of patients-caregivers relationship were examined including the number of multiple responses. For instance, where two diseases were reported in one study, the study was treated twice. Stroke was the most common disease (n = 36; 22.5%), followed by cancer (n = 31; 19.4%) and Alzheimer’s disease (n = 27; 16.9%). In addition, there were studies on the caregivers of patients suffering from mental illness, hemodialysis, brain and spinal cord injury, liver disease, diabetes and Parkinson’s disease. Caregivers were spouses (n = 120; 28.4%), daughter or son of patients (n = 60; 14.2%) and parents of patients (n = 60; 14.2%). In a small number of studies, care was provided from grandchildren, relatives, friends, neighbors, or religious group.

### 3.2 Research Methodology and Conceptual Framework

The majority of studies reported were quantitative studies (n = 111; 76.6%) as 32 studies were qualitative and 2 studies were triangulation studies. Among the quantitative studies, 93 studies employed a non-experimental design, mainly survey. Among the qualitative research studies, a grounded theory approach was mostly common. Among all studies, 35 studies provided a conceptual framework (Table **1**).

### 3.3 Research Trends by Period

We analyzed three periods: 1990–1999 (n = 14, 9.7%), 2000–2009 (n = 69, 47.6%), and 2010–2016 (n = 62, 42.8%). From 2010, more than 10 studies were published each year, and more than 40% of the studies reviewed were published within the past 5 years. The outcome variables used in the target studies were set as indicators and handled by a limited number of multiple responses. After this, their frequency was analyzed according to the decade of publication. Overall, caregivers’ QOL was the topic reported most frequently (58 times), followed by burden (45 times), caregiving experience (36 times) and depression (12 times). During the 1990s, the research topics were confined to QOL, burden, caregiving experience and coping. Beginning in 2000, an increasing number of topics, both “negative” (including stress, depression, and burn out) and “positive” (including well-being, and adaptation) emerged-in all, 22 outcome variables were reported from 2000 to 2009. Support system (*e.g.*, social support) and nursing education (*e.g.*, education needs and program satisfaction) also emerged in studies after 2000, although less frequently. Since 2010, 28 dependent variables have been discussed in studies (Table **2**).

### 3.4 Nursing Theory-Applied Studies

Among a total of 145 studies, 17 (11.7%) studies were theory applied. 17 studies reported a variety of patient diseases, brain diseases being the most significantly reported. Caring was most often provided to a spouse. Nine (52.9%) studies were dissertations, while 8 (47.1%) studies were published in a journal. Of the 17 studies, 15 (88.2%) studies were quantitative. 4 out of the 15 quantitative studies were intervention studies aiming to improve social support through group education. Other studies analyzed caregiving burden, QOL and family function, according to the level of family/social support and nature of the patients–caregivers relationship. In addition, caregiving burden, level of patient function, uncertainty and personality traits were all included as independent variables. QOL was the most commonly studied outcome variable (Table **3**).

The 17 theory-applied studies used a total of 20 theories; of these, six theories (30%) were nursing theories, while the remaining 14 (70%) were theories borrowed from psychology. The most frequently used theory was Lazarus and Folkman’s theory of stress, appraisal and coping [14], followed by House and George’s family caregiver stress cope [15] (Table **4**).

## 4 DISCUSSION

Since 2000, the number of studies on caregivers has spontaneously increased, reflecting the mounting interest in caregivers. In the 1990s, as Korean researchers showed interest in caregiving experiences and changes in their life, specific concepts and attributes of caring emerged. Further, since 2000, the studies on caregiving experiences of caregivers’ QOL have investigated more diverse topics. Outside of Korea, both negative (*e.g.*, burden, decrease in QOL) and positive (*e.g.*, satisfaction, advantage and meaning of caring, compensation) attributes of caregiving experiences have been described [16]. In this study as well, research topics, which were limited to burden, QOL, caregiving experience, and coping in the 1990s, were extended to burnout and depression in 2000s, and more recently, to a broad range of topics, including well-being, posttraumatic growth (PTG), psychological response and caregiving proficiency—thus reflecting more diverse perspectives on caregiving. Likewise, many measurement tools of caregiving experience have emerged since the mid-1990s in other countries [16]. Notably, the development of measures preceded the structure of concepts. Therefore, empirical indicators confirmed that the acquisition of suitable measures played a key role in the expansion of quantitative studies.

In this study as well, caregivers’ QOL was the main focus. We found that the nature of patients–caregivers relationship, caregiver’s well-being, and social support were all the factors positively influencing caregivers’ QOL. Moreover, the relationship between patients and social supports was observed to have a significant effect on caregivers’ QOL. In one study, a group intervention program to improve social support made a positive impact on caregivers’ QOL and reduced caregivers’ burden and stress [17, 18]. Conversely, worsened financial status, increased caring burden, improper caregivers’ physical or mental function, and high level of perceived stress, all negatively influenced caregivers’ QOL. In other words, excessive burden, improper physical health of caregivers and psychological stress were observed to be the negative factors for caregivers’ QOL [19]. Similarly, Grant *et al.* (2013) [20] found that as caregivers’ burden increased, psychological well-being and QOL decreased. Thus, it is urgent to find nursing interventions that can reduce caregivers’ burden and stress and promote emotional well-being.

Similar conclusions were drawn about the caregiving experience. Optimism, caregiving efficacy, social support and quality of patients-caregivers relationship were found to have a positive effect on the caregiving experience. In contrast, disease uncertainty, severity of disease and burden had a negative effect on the caregiving experience [21, 22]. The strong match with the major variables affecting the caregivers’ QOL suggests a close relationship between the caregiving experience and caregivers’ QOL. Therefore, caregivers should be prompted to recognize the value of caring and the satisfaction derived from positive caring experiences to improve their QOL.

The first study on caregivers, published in 1994, was an ethnographic study on the caregivers of cancer patients who were close to death. Since then, there have also been several grounded theory based qualitative studies targeting better understanding of caregiving. The strength of grounded theory qualitative method is that it derives theories on the given phenomena by analyzing empirical data [23]. The early grounded theory studies showed that burden and depression were typical negative attributes of the caregiving experience. Since then, the emergence of diverse concepts has led to the development of a caregiving framework and nursing theories of caregiving. This study investigated burden, QOL, coping, social support and family function, reaching as far back as the 1990s. With the 2000s, there have been continued studies on the negative phenomena, such as burnout, stress and depression; however, increasingly, the focus has been on the experience of positive changes, such as increase in the perception of self-control, satisfaction in overcoming disease and caretaking, and decrease in negative emotions, including depression [5, 6]. Ultimately, reduction in the burden of caring, as well as social support for the improvement of QOL, management of care and positive coping is important [8]. Therefore, nursing studies and practice have emerged to improve those concepts.

Among all the studies, there were very few theory-applied studies. Further, only six studies were driven by nursing theory. In other words, the application of nursing theory to the caregiving experience and caregivers’ QOL has been lacking. Psychological theories have enhanced the understanding of other fields of study, and here as well, there is a need to conceptualize the psychological and emotional aspects of caregiving, such as burden, depression and satisfaction. However, there is a need to develop nursing theories that reflect nursing values. In our review, the most commonly used nursing theories were the Lazarus and Folkman (1984) [14] theory of stress, appraisal, and coping and House and George (1980) [15] theory of family caregiver stress coping. According to these theories, an ultimate goal in nursing [24] is to help patients adapt changes within the societal context, without stress. The focus and goal of nursing studies has been the management and improvement of the caregiving burden and caregiving stress. Caregiving experience and stress related to it and also the stress on caregivers have a negative effect on the patient’s health [24]. Therefore, there is a need for diverse psychological and emotional interventions to decrease caregiving stress and for interventional studies, explaining stress theories based on family role or function, to derive effective and efficient strategies for coping with stress.

According to the research trends for the past 5 years, family unit–oriented concepts, such as “family function,” “family characteristics,” and “family hardiness,” emerged alongside the introduction of caregivers’ well-being and PTG; these did not appear in the studies prior to 2010. In Korea, where the culture of family-centered caring is well developed, the social and moral obligations to care for sick family members are very strong, and caregivers believe themselves to be more important in their family because of their role. The concept of the “family unit” was also described by Suh (2011) [11], who concluded that a holistic approach was needed for caregivers as well as for the patients suffering from disease. One such approach is provided by the Illness Constellation Model [25]. According to this model, the disease experience includes caregiving experience along with that of the patients in which caregivers themselves, along with patients, should be the subjects of care. Thus, in family-unit caring approaches, the patient and caregivers interact. Caregivers should be assisted to recognize the true meaning of caring, through experience and reflection, and helped to adapt to the new demands instead of merely assuming caregiving out of obligation. Further, the establishment of an intervention program and support system among family members reduces the caregiving burden according to personalized needs [9], and allows caregivers’ QOL to be improved by the positive emotions derived from caregiving. Moreover, social support systems must be strengthened, as these can provide human and physical resources (*e.g.*, nursing assistant support, medical benefits, and social network).

PTG [26] refers to the experience of continuous and gradual positive changes through long-term caregiving. In case of Korean caregivers, previous study found the score of PTG to be higher in Korea than in Western countries and attributed this to a Korean emphasis on the quality of care for cancer survivors and for their family members, reflecting the family-centered caring culture in Korean society [27]. These results draw further support for the Illness Constellation Model [25] and provide further proofs that the caregiving experience and caregivers’ QOL can differ depending on the attributes of the patients–caregivers relationship. In addition, spiritual growth appears to be a key element for maintenance of positive ego and life view in the context of caregiving challenges. Thus, psychological and spiritual nursing interventions (considering the caregivers’ personal characteristics) need to be developed.

## CONCLUSION

This study sought to better understand the phenomenon of caregiving through the analysis of Korean studies on the caregiving experience and caregivers’ QOL. We identified a total of 145 studies published from 1990 to 2016 and analyzed the research type, conceptual framework, common patient diseases investigated, patients–caregivers relationship, and the research trends over time. Narrowing the focus of the 17 theory-applied studies and their application of theory, this study aimed to suggest the grounds for the selection of nursing theory appropriate to Korean culture.

The number of studies on the caregiving experience and caregivers’ QOL has increased considerably since the mid-1990s. Certainly, quantitative studies accounted for the greater portion of the studies; however, there were a limited number of intervention studies. In addition, there were relatively few nursing theory-applied studies. Nursing theory provides a foundation for nursing science, practice and education. This study helped presenting empirical data on nursing by identifying the nursing theories applied to the caregiving experience and caregivers’ QOL.

This study is significant in that it contributes to the expansion of nursing knowledge; however, the generalizability of the study findings is limited because only studies on adult caregivers were examined. Furthermore, we used a relatively small number of nursing theories for the analysis, and this limited the ability of nursing theory to explain caregiving. This study focused on the application of nursing theory and identification of the caregivers’ experiences and quality of life; however, the quality assurance of the literature was not assessed because it is not a meta-analysis that compares the effects of interventions on caregivers. When considering the development of nursing interventions for future caregivers, effective intervention and evidence-based nursing practice must be provided through quality assessment and meta-analysis of the literature.

Based on these findings, we offer the following suggestions. First, there is a need to apply the four metaparadigms of “person,” “environment,” “health,” and “nursing” to the investigation of factors affecting the caregiving experience and caregivers’ QOL. Second, there is a need to conceptualize a nursing theory of caregiving that is consistent with the sociocultural context of the Korean caring culture. Finally, nurses caring for older persons should provide theory-based nursing interventions to ameliorate negative psychological or emotional aspects of the caregiving experience and to strengthen the caregivers’ social support system.

## Figures and Tables

**Table 1:** Types of studies and research designs for caregivers in Korea (*N* = 145).

–	–		Total		Conceptual Framework			
					Yes		No	
Research Type	Design	Classifications	n	%	n	%	n	%
Quantitative Research	Experimental Design	True Experimental design	2	1.40	25	17.20	86	59.30
		Quasi-experimental design	4	2.80				
		Pre-experimental design	11	7.60				
	Non- Experimental Design	Systemic literature review	3	2.10				
		Survey	87	60.00				
		Q methodology	2	1.40				
		Case study	1	0.70				
	Others	–	1	0.70				
	Total	–	111	76.60				
Qualitative Research	Grounded theory		18	12.40	9	6.20	23	15.90
	Phenomenological research		5	3.40				
	Narrative research		3	2.10				
	Ethnography research		3	2.10				
	Others		3	2.10				
	Total		32	22.10				
Triangulation	–		2	1.30	1	0.70	1	0.70
Total	–		145	100	35	24.10	110	75.90

**Table 2:** Trend of the selected studies for caregivers in Korea (*N*=145).

Publication Year	1990-1999			2000-2009			2010-2016		
		n	%		n	%		n	%
Outcome Variables	QOL	9	45.00	QOL	25	25.51	QOL	24	23.08
	Burden	7	35.00	Burden	20	20.41	Burden	18	17.31
	CE	3	15.00	CE	17	17.35	CE	16	15.38
	Coping	1	5.00	Depression	5	5.10	Depression	7	6.73
	–	–	–	Stress	4	4.08	Stress	4	3.85
	–	–	–	Burn out	3	3.06	Well-being	3	2.88
	–	–	–	Well-being	2	2.04	Coping	3	2.88
	–	–	–	HRQOL	2	2.04	Adaptation	2	1.92
	–	–	–	Social Support	2	2.04	Health Status	2	1.92
	–	–	–	^*^Others	18	18.37	Social Support	2	1.92
	–	–	–	–	–	–	Anxiety	2	1.92
	–	–	–	–	–	–	Burn out	2	1.92
	–	–	–	–	–	–	^**^Others	19	18.27

Note. CE = caregiving experience; HRQOL = health-related quality of life; QOL = quality of life.

*Others = health-related outcomes; functions; care type; educational needs; perceived health status; needs; mood; relationship with patient; family function; family hardiness; adaptation; sleep; health status.

**Others = family characteristics; caregiver reaction; education needs; suffering experience; program satisfaction; cognitive behavior; conflict process; unmet needs; mental health; psychological response; hope; nursing needs; caregiving mastery; posttraumatic growth; decision making; fatigue.

**Table 3:** Characteristics of the Selected Studies Containing the Theories (*N* = 17).

Year	Authors	Research Type	Participants (n)	Caregivers Type	Disease Type	Theory	Independent Variable	Outcome Variable
1995	Jeong	Quantitative true experimental	44	Not described	brain & spinal cord injuries	T-Double-ABCX Model: Family Stress Theory	Social Support Group	Burden, Quality of life
2000	Hong	Quantitative descriptive survey study	260	Spouse, Son and Daughter, Daughter-in-law, Grandchild, Others	Stroke	A Conceptual Framework for Family Caregiver Burden	Quality of relations, Social support resources, Burden, Well- being, Caring behavior	Well- being, Caring behavior
2000	Kim	Quantitative true experimental	86	Spouse, Son and Daughter, Parents, Siblings	Stroke	Stress-appraisal-coping theory	Support Group Intervention	Various Adaptations
2004	Park	Quantitative true experimental	16	Spouse, Parents	Stroke	Stress-appraisal-coping theory Cognitive-behavior approach model	Support Group Intervention	Stress, Coping way of stress, Satisfaction on life program
2004	Kim	Quantitative true experimental	12	Spouse	Stroke	Rational Emotive Behavior Therapy: REBT	Group program	Quality of life, Rehabilitation motivation Stress Coping
2006	Yu, and Park	Quantitative descriptive survey study	178	Spouse, Others	Stroke	Family caregiver stress cope	Quality of relation, Burden	Family Functioning
2006	Kim	Quantitative descriptive survey study	374	Parents, Spouse, Siblings, Others	Mental illness	Stress-appraisal-coping theory	Social functioning, relationship between a family, Social support to family caregiver	Caregiving experience, Psychological well-being
2008	Kim, and Yu	Quantitative descriptive survey study	124	Spouse, Son and Daughter, Parents, Siblings	Cancer	Family caregiver stress cope	Quality of relations, Performance status	Quality of life
2009	Lee	Quantitative descriptive survey study	303	Spouse, Son and Daughter, Parents, Others	Cancer	Stress-appraisal-coping theory	Social support, Feeling of burden and growth	Quality of life
2011	Choi	Qualitative narrative research	4	Daughter-in-law, Spouse, Son and Daughter	Cancer	Human becoming	Not applicable	Suffering experience
2012	Yoon	Quantitative quasi experimental	10	Spouse, Parents, Son and Daughter	Brain Injury	Transition theory	Transitional Nursing Program	Transition stress, anxiety Burden
2013	Hong, and Tae	Quantitative descriptive survey study	206	Spouse, Parents, Son and Daughter, Others	Cancer	Stress-appraisal-coping theory Family stress theory	Perceived health status, Perceived stress, Hope	Burn out
2014	Kim	Quantitative descriptive survey study	405	Spouse, Parents, Son and Daughter, Others	Chronic Disease	Health Action Process Approach Health Promotion Model	Demographic factor, ^*^Family-care factor ^**^Self-care factor	Self-Care
2014	Choi	Quantitative descriptive survey study	201	Spouse, Parents, Son and Daughter, Others	Cancer	Posttraumatic Growth	Demographic Characteristics, Care related Characteristics	Posttraumatic Growth
2014	Youn, and Tak	Quantitative descriptive survey study	132	Parents, Spouse, Son and Daughter, Siblings, Others	Hemodialysis	Ecological System Theory	Demographic Characteristics	Burden, Social Support, Quality of Life
2015	Oh, and Kim	Qualitative granded theory	200	Not described	Mental illness	Stress-appraisal-coping theory	Optimism, Caregiving self-efficacy, Severity of illness, Uncertainty	Caregiving Experience
2015	La	Quantitative descriptive survey study	102	Spouse, Son and Daughter, Others	multiple myeloma	Stress-appraisal-coping theory	Stress-appraisal	Quality of life

*Family-care factor: caregiving independence level, caregiving time, burden, satisfaction, **Self-care factor: self-care motivation, perceived health action disability, perceived health action usefulness, self-efficient, social support, self-care helper.

**Table 4:** Application of theories on caregivers in the selected studies (*N* = 20).

Variables	Theorists	Year	Theories	n	%
Nursing theories	House, and George	1980	Family caregiver stress cope	2	10.0
	Pallett	1990	A Conceptual Framework for Family Caregiver Burden	1	5.0
	Parse	1991	Human becoming	1	5.0
	Pender	1996	Health Promotion Model	1	5.0
	Meleis	2000	Transition theory	1	5.0
Other disciplines	Lazarus, and Folkman	1984	Stress-appraisal-coping theory	7	35.0
	Hell	1958	Family stress theory	1	5.0
	Meichenbaum	1977	Cognitive-behavior approach model	1	5.0
	Bronfenbrenner	1979	Ecological System Theory	1	5.0
	McCubbin, and McCubbin	1987	T-Double-ABCX Model: Family Stress Theory	1	5.0
	Ellis	1998	Rational Emotive Behavior Therapy: REBT	1	5.0
	Calhoun, and Tedeschi	2004	Post-traumatic growth	1	5.0
	Schwarzer	2008	Health Action Process Approach Model	1	5.0
