# Supplementary material for: A Review of Trend of Nursing Theories related Caregivers in Korea
Source: Open Nurs J. 2018 Feb 8;12:26–35. doi: 10.2174/1874434601812010026 (PMC5824440; doi:10.2174/1874434601812010026)
Supplement: Supplementary file 1 [file TONURSJ-12-26_SD1.pdf]

Send Orders for Reprints to reprints@benthamscience.ae

The Open Nursing Journal, 2018, *12*, i-vi

BENTHAM OPEN

CrossMark

The Open Nursing Journal

Supplementary Material

Content list available at: www.benthamopen.com/TONURSJ/

DOI: 10.2174/1874434601812010026

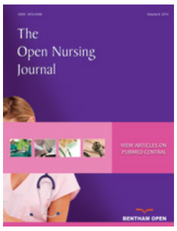

A Review of Trend of Nursing Theories related Caregivers in Korea

SungHae Kim[1], Yoona Choi[1,*], Ji-Hye Lee[2], Da-El Jang[3] and Sanghee Kim[1]

[1]*College of Nursing, Graduate School, Yonsei University, Seoul, Korea*
[2]*College of Nursing, Graduate School, Yonsei University, Samsung medical center, Seoul, Korea*
[3]*College of Nursing, Graduate School, Yonsei University, Severance hospital, Seoul, Korea*

Received: October 13, 2017 | Revised: December 16, 2017 | Accepted: December 25, 2017

Abstract:

Background:

The prevalence of chronic diseases has been rapidly increased due to population aging. As the duration of care needs increase, the caregivers' socioeconomic burdens have also increased.

Objective:

This review examines the attributes of caregiving experience and quality of life of caregivers in Korea with a focus on the application of nursing theory.

Method:

We reviewed studies on caregivers' caring for adult patients published till2016 in 4 bio-medical research portal websites or data bases. A total of 1,939 studies were identified through the keyword search. One hundred forty five studies were selected by a process; of which, 17 studies were theory-applied. Selected studies were analyzed in accordance with the structured analysis format.

Results:

Quantitative studies accounted for 76.6%, while 22.1% were qualitative studies and 1.3% were triangulation studies. Caregiver-related studies increased after 2000. Most frequently, the caregivers were spouses (28.4%), and most frequently, care was provided to a recipient affected by stroke (22.5%). The 17 theory-based studies described 20 theories (70% psychology theories, 30% nursing theories). The most frequent nursing theory was the theory of stress, appraisal and coping.

Conclusion:

This study sought to better understand caregiving through the analysis of Korean studies on the caregiving experience and caregivers' QOL and this finding helped presenting empirical data for nursing by identifying the nursing theories applied to the caregiving experience and caregivers' QOL. The results suggest that the need for further expansion of nursing theories and their greater utilization in the studies of caregiving.

Keywords: Caregivers, Informal caregivers, Nursing theory, Quality of life, Long-term care, Patient care.

INFORMATION OF USING ELECTRONIC DATABASES

Supplementry 1. Literature review in Korea has been commonly used by RISS, NDSL, KISS and DBpia and also those databases provide Korean and English articles.

Electronic Databases	Contents	URI
**RISS**	RISS provides scholarly resources including international and domestic journals/theses.	http://www.riss.kr

Suppl. Table 1 contd.....

Electronic Databases	Contents	URI
**NDSL**	NDSL is the first online DB in Korea including 117,406 journals and provides online databases focusing on science and technology since the 1920s.	http://www.ndsl.kr
**KISS**	KISS provides resources from 4,455 kinds of publications and 1,294 academic journals including KCI, SCI, SSCI, AHCI, and SCOPUS.	http://kiss.kstudy.com
**DBpia**	DBpia provides 2,286 journal databases including 59 medical and 18 nursing journals. Also it contains the field of social science, humanities, engineering, art & sports, medical science, natural science, agronomy, oceanography & fisheries sciences, interdisciplinary studies, and liberal arts.	http://www.dbpia.co.kr

REFERENCE FOR SELECTED STUDIES

Supplementary 2. Literature review in Korea has been commonly used by RISS, NDSL, KISS and DBpia and also those databases provide Korean and English articles.

[1] Ahn DS. A Study on the Perceived Burden and Quality of Life in Family Caregivers with Chronic Liver Disease Patients. Master. diss., Kwandong University 2005.
[2] Ahn EJ. A Study on Burden and Burnout of Family Care-givers for Caring of Terminal Cancer Patients. Master. diss., Chonnam National University 2004.
[3] Baeck HC, Choi YJ. Identifying the Needs of Home Care Patient's Family Caregivers. J Korean Acad Soc Home Care Nurs 2008; 5: 115–21.
[4] Bai JI. The Experience of Families with Psychiatric Patients: A Grounded Theory Approach. J Korean Acad Psychiatr Ment Health Nurs 2001; 10: 53–65.
[5] Bang JS. The burden on the caregiver of a peritoneal dialysis patient and the family hardiness. Master. diss., Inje University 2005.
[6] Chae GJ. Sharing the lived experience of caring for a Spouse with schizophrenia: doing phenomenology. Ph.D. diss., Kang Won University 2013.
[7] Chang JY. A Study of the Attitude toward Withholding Life-Sustaining Treatment and the Characteristics of Cancer Patient Family: Focusing on Do-Not-Resuscitate. Master. diss., Catholic University 2010.
[8] Cheon HJ, Song JA. Family Caregiver’s Experience in Managing Wandering of Elders with Dementia. J Korean Gerontol Nurs 2015; 17: 152–65.
[9] Cho EA. Proposed Theoretical Framework Addressing the Effects of Informal Caregivers on Health-Related Outcomes of Elderly Recipients in Home Health Care. Asian Nurs Res 2007; 1: 23–34.
[10] Cho YS. The Elderly Spouses' Experiences of Providing Care for their Bedridden Patient at home. Ph.D. diss., Kosin University 2015.
[11] Choi ES, Kim KS. Experiences of Family Caregivers of Patients with Terminal Cancer. J Korean Acad Nurs 2012; 42: 280–90.
[12] Choi H, Yoo Y. The Degree of Burden, Coping and Quality of Life in Family Caregivers of Stroke Patients. J Korean Soc Occup Ther 1998; 6: 53–64.
[13] Choi KJ, Back HJ, Kim JE. Burden and Quality of Life in Caregivers of Patients with Rare and Incurable Disease. J Korean Acad Community Health Nurs 2006; 17: 364–75.
[14] Choi KS, Eun Y. A Theory Construction on the Care Experience for Spouses of Patients with Chronic Illness. J Korean Acad Nurs 2000; 30: 122–36.
[15] Choi SN. Family Caregiver Quality of Life: Home health care nursing. Master. diss., Ajou University 2009.
[16] Choi SN. The Research about Health condition, Level of fatigue and Quality of life of the stroke patient's families. Master. diss., Kyung Hee University 2008.
[17] Choi SO. Post traumatic Growth in Family Caregivers of Patients with Cancer. Korean J Hosp Palliat Care 2014; 17: 1–9.
[18] Choi YS. Relationship between Death Orientation of First Care Giver and Quality of Life of Hospice Patients. Master. diss., University of Gachon medicine and science 2006.
[19] Choi YS. Suffering Experience of the Family with Cancer Patients. Master. diss., Konyang University 2011.
[20] Chu MS, Kim HY. The Caregiver's Experience for the Chronic Illness Patient. J Korean Assoc Qual Res 2003; 4: 41–51.
[21] Chung BY, Park HS. Impact Factors for Health of Family Caregivers of Hospice Patients. Korean J Hosp Palliat Care 2014; 17: 75–84.
[22] Chung SH, Yoo YK. A Study on the perceived Burden and the Quality of life of Family Caregivers of Hemodialysis Patients. Korean J Adult Nurs 1997; 9: 324–39.
[23] Eo JM. A Study on the Burden and Life Quality of Cancer Patients' Family Members. Master. diss., Hanyang University 2004.
[24] Gwak YO, Kim YS, Suh KH. Factors of Stress Responses and Quality of Life among Stroke Patients' Family Caregivers: Focused on ADL and Social Supports. Korean J Stress Res 2008; 16: 261–69.
[25] Han JH, Park YH. Caregiver's Burden and Quality of Life of Male Spouses with Stroke Wives. Korean J Adult Nurs 2012; 24(6): 615–26.
[26] Han KH, Jung JG, Oh SK, Kim JS, Kim SS, Kim SY. Depression Level among Family Caregivers of Terminal Cancer Patients. J Korean Acad Fam Med 2005; 26: 752–8.
[27] Han KS, Khim SY, Lee SJ, Park ES, Park YJ, Kim JH, et al. Family Functioning and Quality of Life of the Family Care-giver in Cancer Patients. J Korean Acad Nurs 2006; 36: 983–91.
[28] Han SJ. Factors Influencing Quality of Life of Home Care Patient's Primary Caregiver. J Korean Acad Soc Home Care Nurs 2010; 17: 144–55.

Suppl. Table 2 contd.....

[29] Han YR. The Relationship among Family Primary Caregiver Characteristics, Burden with Hospice Care and Quality of Life. Master. diss., Chungnam National University 2010.
[30] Hong JJ. Prediction Model of Well- Being and Caring Behaviors in Main Caregivers of Patients with Stroke. Ph.D. diss., Hanyang University 2000.
[31] Hong MJ. Structural Relationship of Burnout and Related Variables among Family Caregivers of Cancer Patients. Ph.D. diss., Kosin University 2012.
[32] Hong SW, Son HM. Family Caregivers' Experiences Utilizing a Nursing Home for Their Elderly Family Members. J Korean Acad Nurs 2007; 37: 724–35.
[33] Jang HY, Yi MS. Effects of Burden and Family Resilience on the Family Adaptation of Family Caregivers of Elderly with Dementia. Korean J Adult Nurs 2013; 25: 725–35.
[34] Jang JH. A study on the Caregiver Reaction Assessment(CRA) for Stroke Patient's family caregivers. Master. diss., Korea National Open University 2015.
[35] Jeon HS. Experience of hepatocellular carcinoma patients receiving hepatic arterial chemoembolization and their families. Master. diss., Hanyang University 2014.
[36] Jeong CJ. The Effect of a Caregiver Social Support Group on Burden and Quality of Life of Primary Caregivers with Brain and Spinal Cord Injuries. Korean J Nurs Query 1995; 4: 124–44.
[37] Jeong HJ, Cho BH. A Study of the Effects of Follow-up Care for Stroke Survivors on Primary Caregivers' Quality of Life. Korean J Rehabil Nurs 2000; 3: 169–80.
[38] Joo JL, Park OJ. A Study on the Quality of life and perceived Family support in Dialysis patients. Korean J Adult Nurs 1996; 8: 16–28.
[39] Joo KB. Construction on Primary Care-giver's Burn Out Model of the Senile Dementia Elderly. Ph.D. diss., Kyung Hee University 2009.
[40] Jun JY, Song GS. Burden and Quality of Life in Family Caregivers of Diabetic Inpatients. JKDAS 2006; 8: 2205–19.
[41] Jung HJ. A Phenomenological Study on the Experience of Family Caregivers of Long-term Bed Ridden Elderly Patients. Master. diss., Catholic University of Pusan 2010.
[42] Jung JG, Kim JS, Kim SS, Kang DS, Kim SM, Lee DH, Han KH. Quality of life among Family Caregivers of Terminal Cancer Patient. Korean J Hosp Palliat Care 2006; 9: 1–10.
[43] Kang SJ, Lee HJ, Choi SM. Caregivers' Burden in patients with acute stroke. Korean J Rehabil Nurs 2002; 5: 27–37.
[44] Kang TW, Yeun EJ, Jeon MS. The Burden Types of Primary Care-givers for a Demented Elderly Using Q Methodology. Journal of KSSSS 2014; 28: 53–74.
[45] Kim BL. The Effect of Support Group Intervention on Various Adaptations of Primary Family Caregivers Caring for Cerebro-Vascular Accident Patients. Korean J Adult Nurs 2000; 12: 334–44.
[46] Kim CM, Oh JJ, Choi JM. The Coping Experience of Family Caregivers for the Industrial Disaster Victim. Korean J Occup Health Nurs 2007; 16: 119–29.
[47] Kim CM. The Coping Process of Family Caregivers for Demented Elderly. J Korean Acad Community Health Nurs 2005; 16: 249–59.
[48] Kim DW, Bae ES. Factors Affecting Caregiver Burden in Caregivers of Patients with Parkinson's Disease. Korean J Adult Nurs 2015; 27: 283–93.
[49] Kim EY, Shin EY, Kim YM. Caregiver Burden and Health-related Quality of Life Among Stroke Caregivers. J Korean Acad Community Health Nurs 2004; 3: 5–13.
[50] Kim EY, Yeo JH. Factors associated with Health-related Quality of Life among Family Caregivers of Elders Receiving Home Care Services. J Korean Acad Community Health Nurs 2012; 23: 117–26.
[51] Kim HH. The quality of life and burden by family function among caregiver with chronic patient. Master. diss., Eulji University 2008.
[52] Kim HJ. Relationship between unmet needs and quality of life of family caregivers of cancer patients in Korea. Master. diss., Seoul National University 2012.
[53] Kim HM, Jang GJ. A Study on the Degree of Burden and Quality of Life in Family Caregivers of Patients with Stroke. J Korean Acad Soc Nurs Educ 1998; 4: 81–94.
[54] Kim HO. A Study on Caring behaviors and burden of family caregiver of home care patients with chronic disease. Master. diss., Hanyang University 2011.
[55] Kim HR. A Study on Burden of Stroke Patients' Primary Guardians. Master. diss., Hanyang University 2007.
[56] Kim HS, Yu SJ. Factors Influencing Family Functioning in Family Caregivers of Patients with Cancer. J Korean Acad Fundam Nurs 2008; 15: 301–11.
[57] Kim IJ. Sources of Anxiety and Burden of Family Caregivers of Stroke Patients: The Role of Self-efficacy and Knowledge about Care. Korean J Adult Nurs 2012; 24: 1–10.
[58] Kim IK. A Study of burden and quality of life for family with schizophrenia. Master. diss., Kongju national University 2010.
[59] Kim JE. A Study on the Classification of Self-Care among the Elderly Family Caregivers. Ph.D. diss., Chungnam National University 2014.
[60] Kim JM. The Development of a Group Therapy Program for Stroke Patients and their Families and its Effect. Master. diss., Yonsei University 2005.
[61] Kim JS, Kim MS, KIM SO, Yoo YJ, Won DY. Factors Influencing Dementia Caregivers' Health-related Quality of Life. J Korean Acad Community Health Nurs 2007; 18: 232–41.
[62] Kim K, Han S. Affecting Factors on the Quality of Life of Family Care-givers Caring for the Patients with Cerebral Infarction. Korean J Health Promot Dis Prev 2005; 5: 64–72.
[63] Kim KB, Lee KH. The Correlation Among Health Status, Burden and Quality of Life of the Adult Stroke Patient's Family and the Elderly Stroke Patient's Family. J Korean Acad Adult Nurs 2001; 13: 262–76.

Suppl. Table 2 contd.....

[64] Kim KS, Choe MA, Hah YS, Yi M, Kim BJ, Kim SR, Kim KH, Kwon SH, Hwang YR. Depression and Quality of Life among Family Caregivers of Patients with Parkinson Disease in South Korea. Korean J Rehabil Nurs 2007; 10: 90–8.
[65] Kim MA, Ryu EJ, Hong YP. Caring for Dying Patient with Glioblastoma Multiforme: A Narrative Analysis of the Caring Experience of Family Caregiver. Asian Oncol Nurs 2012; 12: 186–93.
[66] Kim MA. Burden on family caregivers of senile hemodialysis patient. Master. diss., Hanyang University 2015.
[67] Kim MK. The Care Giving Burden of Primary Caregiver based on Nursing Needs of Long-term Care Service User at Home. Master. diss., Woosuk University 2013.
[68] Kim MS, Shin HI, Min Y, Kim JY, Kim JS. Correlation between severe ALS patient-caregiver couples' characteristics and caregivers' health related quality of life. J Korean Acad Nurs 2011; 41: 354–63.
[69] Kim OH. Caregiver's burden and educational needs of acute stroke patient. Master. diss., Eulji University 2009.
[70] Kim SY, Kim JM, Kim SW, Kang HJ, Lee JY, Shin IS, et al. Factors Associated with Quality of Life of Family Caregivers in Terminally Ill Cancer Patients. J Korean Soc Biol Ther Psychiatry 2014; 20: 227–37.
[71] Kim SH. A Study of The Burden, Mental Health and Quality of Life in Family Caregiver with Depressed patient. Master. diss., Ewha Womans University 2010.
[72] Kim SH. An Ethnographic Study on the Caring Process of the Elderly Patient with Chronic Disease by Family. The Journal of Cheju Halla University 2001; 25: 25–39.
[73] Kim SN, Shin SH, Kim MY. The study of experiences in family with person who chronic mental health disorders and degree of satisfaction on day-care center programs. Kum Gu Non Chong 2002; 9: 22–33.
[74] Kim SY. The factors influencing the quality of life in families of patients with liver transplantation. Master. diss., Inje University 2015.
[75] Kim YB, Lee JS. A Study of Relationship between Burden and Quality of Life for Family with Schizophrenia. J Korean Acad Psychiatr Ment Health Nurs 1999; 8: 458–77.
[76] Kim YS. A study on caregiving appraisal and psychological well-being of family caregivers of a person with mental disorder. Ph.D. diss., Ewha Womans University 2005.
[77] Kim YS. A Study on the Relationship between Social Support, Burden and Quality of Life in Intensive Care Unit Patient's Family. Master. diss., Chosun University 2006.
[78] Kim YS. The Effects of Characteristics of Caregiving Situation, Coping Strategies, Social Support on Depression of Family Caregiver of the Demented Elderly. Master. diss., Pusan National University 2002.
[79] Kong EH. Family Caregivers of Older People in Nursing Homes. Asian Nurs Res 2008; 2: 195–207.
[80] Kwag YK. Effectiveness of a Telephone Coaching for the family burden of the caregiver of the schizophrenia in home. Master. diss., Yonsei University 2004.
[81] Kwon EJ, Yi MS. Husbands' Caring Experiences for Women with Breast Cancer in Korea. Asian Oncol Nurs 2013; 13: 18–27.
[82] Kwon HK, Song M. Role Adaptation Processes of Family Caregivers with Patients Transferred from Intensive Care Unit to General Ward: Becoming almost a Nurse with Hope and Fear. Korean J Adult Nurs 2014; 26: 603–13.
[83] Kwon S, Tae YS. The Experience of Adult Korean Children Caring for Parents Institutionalized with Dementia. J Korean Acad Nurs 2014; 44: 41–54.
[84] Kwon SH, Tae YS. Nursing Home Placement: The Process of Decision Making and Adaptation among Adult Children Caregivers of Demented Parents in Korea. Asian Nurs Res 2012; 6: 143–51.
[85] La IS. Effects of stress appraisal on the quality of life of adult patients and their primary family caregivers with multiple myeloma: Testing dyadic dynamics using Actor-Partner Interdependence Model. Master. diss., Kyung Hee University 2015.
[86] Lee EJ, Kim HD. A Study of the Relation between Quality of Life and Family Burden of Home-based Hospice Patient Families. Korean Journal of Hospice Care 2006; 6: 69–78.
[87] Lee HJ, Kim KR, Seo JM. Effects of Telephone Counseling on Burnout, Depression, Life Satisfaction, and Percieved Health in Caregivers of older Adults with Dementia. J Korean Acad Nurs 2003; 15: 452–62.
[88] Lee HJ, Singh J. Appraisals, Burnout and Outcomes in Informal Caregiving. Asian Nurs Res 2010; 4: 32–44.
[89] Lee HJ. A Burden of the family care-givers for stroke patients at home. Master. diss., Yonsei University 1999.
[90] Lee HJ. A Case study on the experiences of liver transplant recipients and their spouses. Master. diss., Yonsei University 2000.
[91] Lee HM. A Study on the Stress, Nursing Needs, and Quality of Life of the Family Caregiver who has family member with Cancer under Chemotherapy in outpatient clinics. Master. diss., Hanyang University 2011.
[92] Lee HY. A Correlation Study of Burden and Quality of Life in Family Caregiver of Dementia Patient. Master. diss., SahmYook University 1997.
[93] Lee JS. The Development & Evaluation of the Caregiving Stress Management Program for Families of Terminal Cancer Patients. Ph.D. diss., Korean University 2006.
[94] Lee KL. The Effects of Supportive Nursing Care Providing Emotional Care & Information on the Stress & Care Skills of Active Brain Stroke Patients` Families. Master. diss., Chonnam National University 2009.
[95] Lee KO, Yang HY. Family Caregiver Burden and Quality of Life According to the Levels of CVA Patient's Self-Care. J Korean Acad Psychiatr Ment Health Nurs 2002; 11: 192–205.
[96] Lee KO. A Study on Quality of life and It's Influencing Factors in Family Caregivers Caring for Dementia Patient. J Korean Acad Psychiatr Ment Health Nurs 2003; 12: 15–26.
[97] Lee KR. The Caring Lived Experience of the Adulthood Stroke Patients' Families. Master. diss., Kyung Hee University 2001.
[98] Lee KS. A Study on the Family's Caregiving Experience in the Dementia. J Korean Acad Psychiatr Ment Health Nurs 1996; 5: 50–8.

Suppl. Table 2 contd.....

[99] Lee KS. Wives' Caregiving Experience with Absolute bed Rest Husband in the Hospital. J Korean Acad Psychiatr Ment Health Nurs 2003; 12: 552–64.
[100] Lee MH. The Effects of the Structured Dementia Education on the Appraisal of Caregiving Experiences by the Main Family Caregivers of Dementia Patients and on those Patients' Cognitive Behaviors. Master. diss., Dong Eui University 2011.
[101] Lee OK. A Study on Caregivers Burden of Elderly Stroke Patients. Master. diss., Gongju National University 2004.
[102] Lee TY. Effects of Supportive Education Program for Hospice Patients' Families. Master. diss., Keimyung University 2012.
[103] Lee YA. Adaptation Process of The Family with Hemodialysis. Korean J Rehabil Nurs 1999; 2: 184–92.
[104] Min YS, Yoong JS. Relationships Among Burden, Social Support and Quality of Life for the Family Caregiver of Hemodialysis Patients. J Korean Acad Psychiatr Ment Health Nurs 2003; 12: 358–66.
[105] Na DM, Chung Y, Yang KM. Burden and Quality of Life in Main Caregivers of Cancer Patients Treated with Chemotherapy. J Korean Acad Community Health Nurs 2003; 14: 1–13.
[106] Na YR. A Study on the Caregiver's Burden and the Intention of Using the Long-term Care Facilities for in the Elderly Patients with Hemodialysis. Master. diss., Ewha Womans University 2006.
[107] Oh HJ. Psychosocial Responses and Quality of Life among Amyotrophic Lateral Sclerosis Patients and Their Caregivers. Korean J Rehabil Nurs 2011; 14: 103–10.
[108] Oh IO, Kim S. Structural Equation Model for Caregiving Experience of Families Providing Care for Family Members with Mental Disorders. J Korean Acad Nurs 2015; 45: 97–106.
[109] Oh J, An JW, Oh KW, Oh SI, Kim JA, Kim SH, Lee JS. Depression and caregiving burden in families of patients with amyotrophic lateral sclerosis. J Korean Acad Nurs 2015; 45: 202–10.
[110] Oh JJ. A Comparative Study on the Effects of an Individual Intervention Program and a Group Intervention Program on the Demented Elderly and Their Families. J Korean Acad Community Health Nurs 2008; 19: 205–15.
[111] Park ES, Lee SJ, Park YJ. A Model for Quality of Life of Family Caregivers with a Chronically Ill Patient. J Korean Acad Nurs 1998; 28: 344–57.
[112] Park HJ. A Study on the Effect of Group program to manage Stress of Family Members of Cerebral Apoplexy Patients. Ph.D. diss., Ewha Womans University 2004.
[113] Park JY. Relationship between Stress and Quality of Life of Family Caregivers of Patient with Lung Cancer. Asian Oncol Nurs 2010; 10: 129–36.
[114] Park MN. Function of Social Support on Quality of Life for the Family Caregiver of Chronic Inpatient. Master. diss., Chonnam National University 2005.
[115] Park MY. The Relationship between Burden, Spiritual well-being, and Quality of life in family of terminal cancer in patients in Korea hospice palliative care institutions. Master. diss., Kosin University 2015.
[116] Park SJ. A Prediction Model for the Quality of Life of Spouses of In-Home Care Elderly with Dementia. Ph.D. diss., Chung-Ang University 2015.
[117] Park SY. The Experience of Husband caring for Wife with Early-Onset. Ph.D. diss., Hanyang University 2013.
[118] Rhee YS. A Study of Effect on Quality of life of Cancer patient's Caregiver: Focusing on the Mediating effect of Feeling of burden and Growth. Ph.D. diss., Ewha Womans University 2009.
[119] Ro YJ, Kim CG. A Comparison of Quality of Life between the Families of Hospital Hospice Patients and Those of Home-Based Hospice Patients. J Korean Acad Nurs 1998; 28: 773–85.
[120] Roh ES, Kwon HJ, Kim KH. A Study on Caring Experiences of the Families of the Seriously Ill Patients. J Korean Acad Nurs 1997; 9: 251–61.
[121] Seo HH, Hah YS. The Effect of Family Psychoeducation on Knowledge and Caregiving Experience for the Family with Schizophrenic Patient. J Korean Acad Psychiatr Ment Health Nurs 2003; 12: 565–75.
[122] Seo IS. The Experiences of Daughters-in-Law Taking Care of their Demented Mothers-in-Low: Narrative Inquiry. Ph.D. diss., Ewha Womans University 2003.
[123] Seomun J, Jung Y. A Qualitative Study on Caregivers' Burden Experiences for the Long-term Care Qualified Elderly. Journal of Institute for Social Sciences 2011; 22: 3–30.
[124] Song EJ, Jo HK. Effects of 'Happy Plus Program' for Family Caregivers of Patients with Demenia on Sleep, Depression, Burden, and Quality of Life of Caregivers. J Korean Acad Psychiatr Ment Health Nurs 2014; 23: 49–59.
[125] Song JA, Park JW, Chun HJ, Park MH. Development of Web-based Educational Program for Family Caregivers in Managing Behavioral and Psychological symptoms of Dementia. Journal of the Korean Gerontological Society 2015; 35: 411–32.
[126] Song JM. Burden and Quality of Life of Family Caregivers in Spinal Cord Injury. Master. diss., Hanyang University 2011.
[127] Song MR. Study on the relationship between pulmonary tuberculosis patients' family support and their quality of life. Master. diss., Kongju national University 2016.
[128] Song SE. A Study on the Health Conditions and Education Needs for Family of the Stroke Patients. Master. diss., Ewha Womans University 2012.
[129] Suh SR, Seo JY, Choi EH, Park WJ. The Effects of Aromatherapy on Depression, Sleep, Mood, and Smell in Stroke Patients and Their Caregivers. J Korean Acad Community Health Nurs 2008; 19: 27–35.
[130] Sung IS, Kim JY, Noh GO, Ahn KD, Ryu EJ, Kwon IG. Quality of Life and Family Burden in Cancer Patients. Korean J Adult Nurs 2007; 19: 603–13.
[131] Sung MR, Yi M, Lee DY, Jang HY. Overcoming experiences of family members caring for elderly patients with dementia at home. J Korean Acad Nurs 2013; 43: 389–98.

Suppl. Table 2 contd.....

[132] Won JS. An Ethnographic study of caring for dying patient with cancer. Ph.D. diss., Ewha Womans University 1994.
[133] Woo YJ. Caregiver Burden, Coping and Quality of Life according to Gender among Spouses of Cancer Patients Undergoing Chemotherapy. Master. diss., Seoul National University 2015.
[134] Yang EY, Kim YA. Burden and Quality of Life in Terminal Cancer Patient's Family Caregivers in the area of Jeollanam-do. J Korea Acad Industr Coop Soc 2015; 16: 3954–62.
[135] Yang WJ. A Study of Influencing Factors on Quality of Life of Family Caregivers of Patients with Lung Cancer. Master. diss., Yonsei University 2007.
[136] Yih BS, Kim CM, Yi MS. Women Caregivers' Experiences in Caring at Home for a Family Member with Dementia: A Feminist Approach. J Korean Acad Nurs 2004; 34: 881–90.
[137] Yoo JS, Lee JH, Chan SJ. Family Experiences in End-of-Life Care: A Literature Review. Asian Nurs Res 2008; 2: 223–34.
[138] Yoo MS, Kim YS, Kim KS. A Study about Health related Quality of Life, Burden and Coping Ability for Family Caregivers caring for Dementia Elderly. Journal of the Korean Gerontological Society 2010; 30: 1117–27.
[139] Yoon SH. Development and Evaluation of Transitional Nursing Program of Brain Injury Patients and Family Caregivers in Neurological Care Unit. Ph.D. diss., Yonsei University 2012.
[140] Yoon SK. Burden, social support and quality of life of the family caregivers of hemodialysis patients. Master. diss., Hanyang University 2014.
[141] Yu HY, Shin JI. Correlation between Depression of Stroke Caregivers and Their Life Quality. The Journal of Korean Association Occupational Therapy Policy for Aged Industry 2011; 3: 59–66.
[142] Yu SJ, Park YH. Influencing Factors on Family Functioning of Caregivers in Families with Stroke Patients. Korean J Adult Nurs 2006; 18: 457–67.
[143] Yun JW. The relevance research of depression and anxiety and coping strategies for primary caregiver of stroke patients. Master. diss., Dongguk University 2015.
[144] Yun MH, Choi SM. Quality of Life and the Factors Related to Family Caregivers Caring for Those with Amyotrophic Lateral Sclerosis. Perspect Nurs Sci 2011; 8: 62–72.
[145] Yun SH. Types of caring for family caregivers of patients with terminal cancer. Master. diss., Hanyang University 2008.

© 2018 Kim *et al*.

This is an open access article distributed under the terms of the Creative Commons Attribution 4.0 International Public License (CC-BY 4.0), a copy of which is available at: https://creativecommons.org/licenses/by/4.0/legalcode. This license permits unrestricted use, distribution, and reproduction in any medium, provided the original author and source are credited.
